# Nature’s magic: how natural products work hand in hand with mitochondria to treat stroke

**DOI:** 10.3389/fphar.2024.1434948

**Published:** 2025-01-07

**Authors:** Lin Cheng, Shangbin Lv, Chengkai Wei, Sucheng Li, Hao Liu, Yong Chen, Zhaoliang Luo, Hongyan Cui

**Affiliations:** ^1^ Department of Neurology, Chongqing Kaizhou Hospital of Traditional Chinese Medicine, Chongqing, China; ^2^ Chongqing Universty of Traditional Chinese Medicine, Chongqing, China; ^3^ Department of Encephalopathy, Chongqing Traditional Chinese Medicine Hospital, Chongqing, China; ^4^ Department of Rehabilitation Medicine, The Fifth People’s Hospital of Chongqing, Chongqing, China

**Keywords:** mitochondria, stroke, ischaemic injury, natural products, neuronal protection

## Abstract

**Background:**

Mitochondria, as the energy factories of cells, are involved in a wide range of vital activities, including cell differentiation, signal transduction, the cell cycle, and apoptosis, while also regulating cell growth. However, current pharmacological treatments for stroke are challenged by issues such as drug resistance and side effects, necessitating the exploration of new therapeutic strategies.

**Objective:**

This review aims to summarize the regulatory effects of natural compounds targeting mitochondria on neuronal mitochondrial function and metabolism, providing new perspectives for stroke treatment.

**Main findings:**

Numerous *in vitro* and *in vivo* studies have shown that natural products such as berberine, ginsenosides, and baicalein protect neuronal mitochondrial function and reduce stroke-induced damage through multiple mechanisms. These compounds reduce neuronal apoptosis by modulating the expression of mitochondrial-associated apoptotic proteins. They inhibit the activation of the mitochondrial permeability transition pore (mPTP), thereby decreasing ROS production and cytochrome C release, which helps preserve mitochondrial function. Additionally, they regulate ferroptosis, mitochondrial fission, and promote mitochondrial autophagy and trafficking, further enhancing neuronal protection.

**Conclusion:**

As multi-target chemical agents, natural products offer high efficacy with fewer side effects and present promising potential for innovative stroke therapies. Future research should further investigate the effectiveness and safety of these natural products in clinical applications, advancing their development as a new therapeutic strategy for stroke.

## Introduction

Stroke, also known as cerebrovascular accident (CVA), is a sudden onset condition caused by an interruption in the blood supply to the brain or by hemorrhage, leading to localized ischemic or hemorrhagic injury and subsequent brain function impairment (Campbell and Khatri, 2020; Campbell et al., 2019). Strokes are classified into ischemic stroke and hemorrhagic stroke (Feske, 2021). Ischemic strokes are predominant, accounting for approximately 80%–85% of all stroke cases, while hemorrhagic strokes constitute the remaining 15%–20% (Boursin et al., 2018). Current treatment strategies for stroke include acute phase interventions and rehabilitation therapies (Caprio and Sorond, 2019). However, these methods are limited by narrow therapeutic windows and carry risks such as increased bleeding (Teasell et al., 2020). Thus, there is a critical need in clinical practice for new, safer therapeutic approaches.

Mitochondria are intracellular organelles commonly referred to as the “powerhouses of the cell,” responsible for energy production within cells (Nunnari and Suomalainen, 2012). They consist of a double-membrane structure, with a relatively smooth outer membrane and an inner membrane that forms numerous folded invaginations known as cristae (Frey and Mannella, 2000). These cristae increase the surface area of the inner membrane, enhancing mitochondrial energy production efficiency. Mitochondria generate ATP through oxidative phosphorylation, a process involving the oxidation of organic molecules like glucose, releasing energy, and storing this energy in ATP molecules (Schatz, 1967). Beyond energy production, mitochondria have other crucial functions, including maintaining intracellular calcium ion balance, regulating apoptosis, participating in cellular signal transduction, and modulating cellular metabolic pathways (Faas and de Vos, 2020; Du et al., 2016; Si et al., 2024). Currently, research on targeting mitochondria for disease treatment spans multiple fields, including neurological disorders, cardiovascular diseases, and metabolic diseases (Geng et al., 2023; Chistiakov et al., 2018; Bhatti et al., 2017). In the realm of neurological disorders, stroke is a significant focus of research. Since mitochondria can enhance cell tolerance and reduce cell death, thereby protecting against neuronal damage, targeting mitochondria presents a promising therapeutic approach for stroke.

Natural products refer to organic compounds derived from nature, including compounds produced by plants, animals, and microorganisms (Wilson et al., 2020). These compounds typically have good biocompatibility and safety profiles, with fewer severe side effects. They often exhibit various biological activities, such as antibacterial, anti-inflammatory, antioxidant, and antitumor effects, making them valuable for drug discovery and the development of new therapeutic methods (Crane and Gademann, 2016; Grigalunas et al., 2022). Natural products usually have multi-target characteristics, affecting multiple biological processes simultaneously, including mitochondrial functions (Mu et al., 2020). Therefore, they can modulate multiple aspects of mitochondrial activity, thereby more comprehensively influencing the occurrence and progression of stroke. Currently, research on natural products for stroke treatment is still in its early stages but has shown some potential. Some natural products have been found to possess antioxidant and anti-inflammatory properties, which can mitigate neuroinflammatory responses and cellular damage following a stroke. Examples include tea polyphenols, flavonoids, and curcumin (Tressera-Rimbau et al., 2017; Li R. et al., 2023; Ran et al., 2021). These compounds help protect neurons from stroke damage by reducing oxidative stress and inflammatory responses. Stroke is a complex disease involving damage to various cell types, including neurons and glial cells (Blacker, 2003). Natural products may offer protective effects for multiple cell types, helping to maintain the integrity of brain tissue.

In the treatment of stroke, although existing therapies such as thrombolytic therapy and antiplatelet drugs can alleviate acute-phase symptoms to a certain extent, their limitations are still significant, especially in terms of the time window of treatment, the side effects of the drugs, and the individual differences in outcomes (Teasell et al., 2020). In recent years, therapeutic strategies in which natural compounds target mitochondria have attracted much attention (Wilson et al., 2020). Mitochondria are the energy factories of cells and are involved in cellular energy metabolism, signalling, oxidative stress response and apoptotic processes (Nunnari and Suomalainen, 2012). During the acute phase of stroke, brain tissue suffers from ischaemia, oxidative stress, inflammatory response and other injuries, and the damage and dysregulation of mitochondrial function is an important cause of brain cell death. Natural compounds are particularly suitable for intervening in stroke therapy by protecting mitochondrial function due to their multi-targeting, low toxicity and good biocompatibility. Therefore, studying and summarising how natural products target mitochondria for stroke treatment may become one of the important strategies for the treatment of stroke.

## Association of mitochondria with stroke

Mitochondria play an important role in the pathogenesis of stroke, including ischaemia-reperfusion injury, apoptosis, and oxidative stress (Yang et al., 2018). Brain tissue has a very high demand for oxygen and energy to maintain normal neuronal function and signalling. When ischaemia occurs in stroke, mitochondrial function is impaired due to hypoxia in brain tissue, resulting in reduced ATP production and disturbed cellular energy metabolism, which can exacerbate brain tissue damage (Ames, 2000). In addition, impaired mitochondrial function leads to increased intracellular Ca^2+^ levels, triggering intracellular calcium overload (Jiang et al., 2023). This triggers a variety of pathological processes, including activation of proteases, disruption of mitochondrial membrane integrity, and opening of the mitochondrial permeability transition pore (mPTP) leading to apoptosis (Li et al., 2020a). Meanwhile, in stroke, hypoxia and ischaemia lead to impaired mitochondrial function, producing more oxygen free radicals and other reactive oxidants (Li Y. et al., 2023). These oxidants attack the cell membrane, proteins and DNA from the damaged mitochondrial membrane as well as mPTP and spill over into the cytoplasm, leading to cellular damage and apoptosis and exacerbating brain tissue damage (Cadenas, 2018). In stroke, damaged mitochondria release apoptosis-associated proteins, such as cytochrome C, which activate the mitochondria-associated apoptotic pathway, leading to apoptosis and neuronal loss in brain cells (Tucker et al., 2018).

In the pathogenesis of stroke, mitochondria not only play a key role in energy metabolism and cell death mechanisms, but also have important functions in maintaining intracellular homeostasis and responding to external stimuli. Stroke leads to an increase in oxidative stress, which subsequently causes cellular damage and inflammatory responses (Reiter et al., 2016). Mitochondria-targeted therapies can mitigate oxidative stress while simultaneously inhibiting the onset and progression of inflammatory responses, thereby reducing the release of inflammatory cytokines and lowering the inflammation levels in brain tissue (Wang et al., 2018). Ferroptosis, a novel cell death mechanism, is characterized primarily by cellular damage and death due to abnormal accumulation of iron ions and exacerbated oxidative stress (Stockwell et al., 2017). Stroke induces oxidative stress and inflammatory responses in brain tissue, increasing the levels of intracellular free iron ions, which in turn triggers ferroptosis (Xu et al., 2023).

Mitochondrial fission refers to the process by which a single mitochondrion divides into two or more smaller mitochondria (Youle and van der Bliek, 2012). This process helps cells regulate mitochondrial quantity, morphology, and function to adapt to the cell’s energy demands and environmental changes (Quiles and Gustafsson Å, 2022). During a stroke, brain tissue is affected by adverse factors such as ischemia and hypoxia, leading to impaired mitochondrial function. Under these conditions, mitochondrial fission may be activated, causing the mitochondria to divide in response to oxidative stress and energy metabolism disturbances (Wu et al., 2022; An et al., 2021). However, these newly generated mitochondria may be unable to adequately produce sufficient ATP, thereby exacerbating the metabolic disturbances in brain tissue and impairing normal neuronal function. Excessive mitochondrial fission can result in neuronal damage and apoptosis, aggravating the pathological progression of stroke (Xu et al., 2017).

Mitophagy is a crucial cellular self-degradation process that clears damaged mitochondria and promotes the synthesis of new ones, thereby maintaining mitochondrial quantity and function within cells (Bravo-San Pedro et al., 2017). Maintaining stable mitochondrial function and morphology is critical for mitigating stroke damage. Stroke induces hypoxia and ischemia in brain tissue, leading to disruptions in cellular energy metabolism. Under these circumstances, mitophagy clears damaged mitochondria, reduces oxidative stress levels, and restores cellular energy metabolism (Guan et al., 2018). Mitophagy also reduces the release of oxidative stress and apoptotic signals, thereby decreasing neuronal apoptosis and brain tissue damage (Zhong et al., 2022). Additionally, when stroke triggers neuronal damage by promoting the release of pathological inflammatory mediators, mitophagy mitigates neuroinflammatory responses by reducing the release of intracellular inflammatory mediators, thus alleviating brain tissue damage (Liu et al., 2023).

**FIGURE 1:**
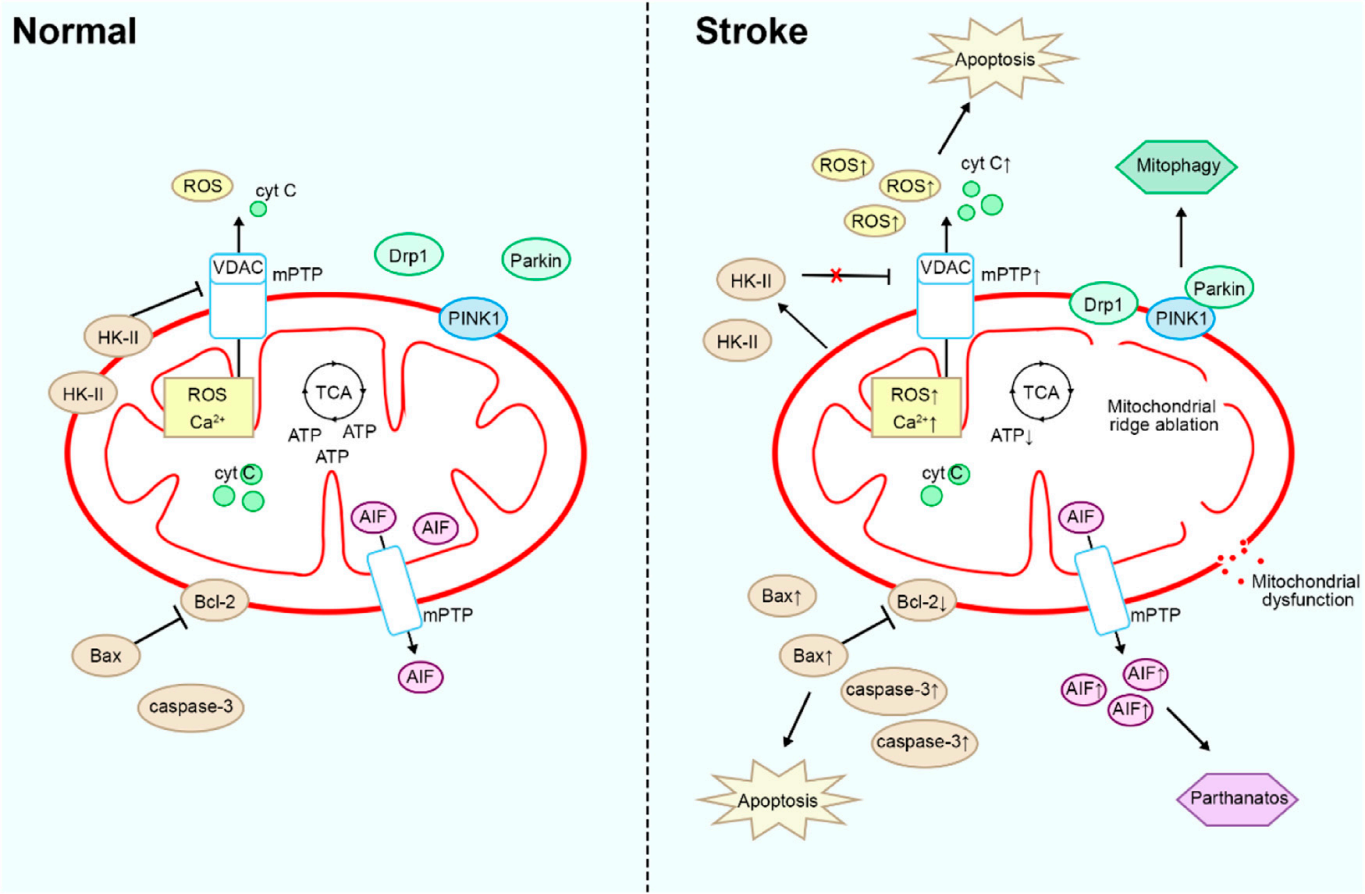
Mitochondrial physiopathological mechanisms in stroke.

In summary, targeting mitochondria can directly impact the pathological processes of stroke, aiding in the prevention or alleviation of brain tissue damage (Figure 1). Natural compounds exert multifaceted effects through multi-target interactions to effectively regulate mitochondrial function, thereby mitigating the damage caused by stroke. These compounds protect mitochondria and reduce oxidative stress and inflammation by diminishing ROS production, inhibiting the opening of the mPTP, and modulating ferroptosis pathways. As a result, they safeguard neurons and alleviate stroke-induced injury. Furthermore, natural compounds can regulate mitophagy, which effectively suppresses the damage caused by excessive mitochondrial fission. Through these diverse mechanisms, natural products provide neuroprotective effects, reduce neuronal injury, and attenuate the pathological processes associated with stroke. However, there has been limited investigation into natural products that regulate mitochondrial pathways in stroke. Therefore, the development of therapeutics targeting mitochondria represents a promising avenue for combating stroke. This review attempts to categorize recent publications on the mechanisms by which natural products exert anti-stroke effects, focusing particularly on the molecular mechanisms involving mitochondria.

## Natural compounds target mitochondria to treat stroke

**FIGURE 2:**
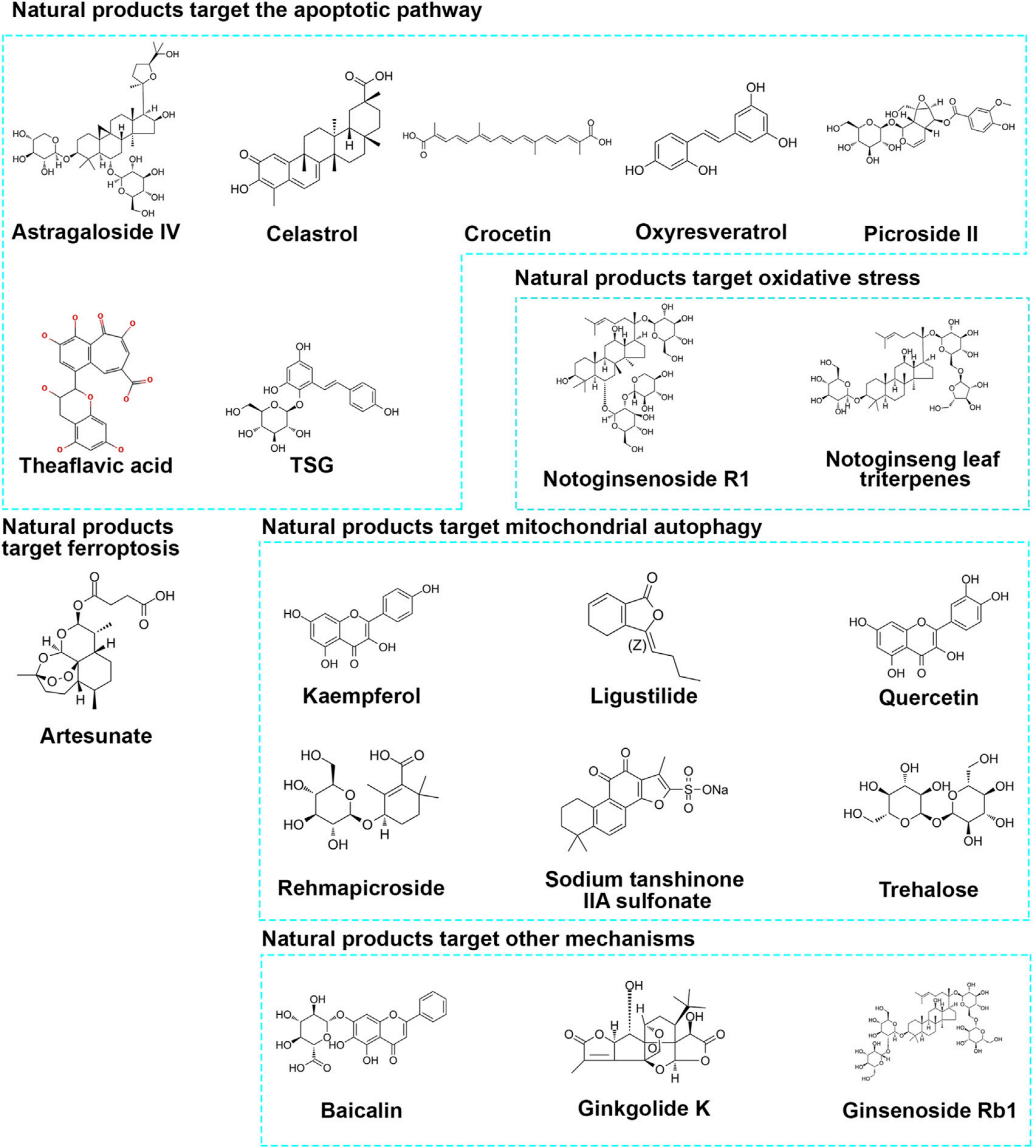
Chemical structure of natural products.

As we screen and summarize natural products that protect neurons through mitochondrial pathways, we have found ample evidence from *in vitro* and *in vivo* studies confirming their ability to target mitochondria and protect neurons. We have classified the pathways targeting mitochondria as follows: 1) targeting mitochondrial apoptosis pathway regulation; 2) targeting oxidative stress regulation of mitochondria; 3) targeting Ferroptosis regulation of mitochondria; 4) targeting mitophagy regulation of mitochondria; 5) other pathways. Please refer to Figure 2 and Table 1 for details.

**TABLE 1:** Molecular mechanisms of natural compounds for stroke treatment by regulating mitochondrial function.

Natural compounds	Species	Animal/cell (dose)	Target for drugs	Reference
Natural product targets mitochondrial apoptosis pathway for stroke treatment				
Astragaloside IV	*Astragalus membranaceus* Schischk.	MCAO/R mice (30 mg/kg, ip, once/d, 7 d) OGD/R cell (10 μM)	Improve mitochondrial dysfunction, p-AKT↑, AKT↓, HK-II↓, AIF↓, ATP↑, NAD+↑, Bax↓, mPTP↑, caspase-3↓, caspase-8↓, cleaved caspase-3↓, Bid↓, Cyto C↓	Li et al. (2019) Yin et al. (2020)
Celastrol	*Tripterygium wilfordii* Hook. f.	ICH mice (2 mg/kg, ip, once/d, 3 d) Primary cortical neuron (10 μM)	Improve mitochondrial dysfunction, mPTP↓, VDAC1↓, EPAC-1↓, mmp↑, ATP↑, ROS↓, Ca^2+^↓	Xue et al. (2019)
Crocetin	*Crocus sativus* L.	MCAO/R rats (40 mg/kg, po, once/d, 5 d) OGD/R SH-SY5Y cell (25 μM)	Improve mitochondrial dysfunction, NOX-2↓, HK-I↓, ROS↓, mmp↑, GSH↑, MDA↓, PARP-1↓, PAR↓, AIF↓, p-p47↓	Li et al. (2024)
Oxyresveratrol	*Veratrum album* L.	MCAO/R rats (20 mg/kg, ip, once/d, 2 d)	Improve mitochondrial dysfunction, Cytochrome C↓, cleaved-caspase-3↓	Hong et al. (2023)
Picroside II	*Neopicrorhiza scrophulariiflora* (Pennell)	MCAO/R rats (20 mg/kg, ip, once, after MCAO/R 2h)	Improve mitochondrial dysfunction, mPTP↓, VDAC1↓, ROS↓, EndoG↓	Wu et al. (2023)
Theaflavic acid	Black tea	OGD/R PC12 cell (50 μM)	LDH↓, ROS↓, Ca2+↓, MDA↓, SOD↑, mmp↑, cleaved-caspase-3↓, Bcl-2/Bax↑, Nrf2↑, HO-1↑	Yoshino et al. (2011)
TSG	*Pleuropterus multiflorus* (Thunb.) Nakai	MCAO/R rats (40 mg/kg, ip, prior to reperfusion) OGD/R cell (25 μM)	ROS↓, Ca^2+^↓, mmp↑, Bcl-2/Bax↑, p-JNK↓, iNOS↓, SIRT1↑	Andrabi et al. (2004)
Natural products target mitochondrial oxidative stress to treat stroke				
Notoginsenoside R1	*Panax notoginseng* (Burk.) F. H. Chen	Sprague-Dawley rats (40 mg/kg, ip, once, after MCAO) Neuro-2a cell (20 μM)	GLUT 1/3↑, MCT↑, ATP↑, mitochondria number↑, mmp↑, mtDNA↑, Atp12a↑, Atp6v1g3↑	Zhang et al. (2017)
Notoginseng leaf triterpenes	*Panax notoginseng* (Burk.) F. H. Chen	MCAO/R rats (292 mg/kg, ig, once/d, 2 weeks) OGD/R SH-SY5Y cell (6.25 μg/mL)	Mitochondrial ridge protection, ATP↑, mmp↑, NAMPT↑, SIRT1/2/3↑, MnSOD↑, PGC-1α↑, Foxo3a↑	Li et al. (2020b) Mu et al. (2021)
Panax notoginseng saponins	*Panax notoginseng* (Burk.) F. H. Chen	Sprague-Dawley rats (60 mg/kg, po, once/d, 6 months)	Improve mitochondrial dysfunction, Mitochondrial ridge protection, Bcl-2/Bax↑, caspase-3↓, FoxO3a↑, Mn-SOD↑, PGC-1α↓, LC3β↓, Beclin-1↓	Wang et al. (2009)
Natural product targets ferroptosis to treat stroke				
Artesunate	*Artemisia caruifolia* Buch.-Ham.	ICH rats (70 mg/kg, ip, once/d, 3 d before ICH) BV2 cell (10 μM)	Ferroptosis↑, p-AMPK, ↑ mTORC1↓, p-Akt↓, GPX4↓, ROS↑, lipid peroxidation↑	Brooks (2018)
Baicalein	*Scutellaria baicalensis* Georgi	MCAO/R mice (80 mg/kg, ip, once/d, 7 d) OGD/R HT22 cell (4 μM)	Ferroptosis↓, Improve mitochondrial dysfunction, GPX4↑, ACSL3↑, ACSL4↓, GSH↑, SOD↑, MDA↓, LPO↓	Liu et al. (2022)
Cottonseed oil	*Gossypium hirsutum* Linn.	MCAO/R rats (1.3 mL/kg, sc, once/2d, 3 weeks)	Ferroptosis↓, Improve mitochondrial dysfunction, GPX4↑, xCT↑, HO1↑, FTH1↑, ACSL4↓, GSH↑, SOD↑, MDA↓, LPO↓	Meng et al. (2014)
Natural product targets mitochondrial autophagy for stroke treatment				
Kaempferol	*Brassica capitata* var. *italica*	MCAO/R rats (200 mg/kg, po, once/d, 7 d) OGD/R cell (10 μM)	Mitophagy↑, Improve mitochondrial dysfunction, p-Akt↓, p-Drp1↑, HK-II↑, LC3↑, ROS↓	Yan et al. (2021)
Ligustilide	*Angelica sinensis* (Oliv.) Diels	MCAO/R rats (20 mg/kg, ip, once/d, 3 d) OGD/R HT-22 cell (20 μM)	Mitophagy↑, Improve mitochondrial dysfunction, PINK1↑, Parkin↑, TOMM20↑, LC3↑, ROS↓	Bai et al. (2016)
Quercetin	*Glycyrrhiza uralensis* Fisch.	MCAO/R rats (5 mg/kg, iv, once, 2 h after surgery) BMECs (200 μM)	Mitophagy↑, Improve mitochondrial dysfunction, p-Akt↓, p-mTOR↓, HIF-1α↓, PINK1↑, Parkin↑, LC3↑, TFEB↓, ROS↓	Xie et al. (2020)
Rehmapicroside	*Rehmannia glutinosa* (Gaert.) Libosch	MCAO/R rats (10 mg/kg, ip, once, at the onset of reperfusion) OGD/R SH-SY5Y cell (50 μM)	Excessive mitophagy↓, Inhibition of mitochondrial autophagy, Bcl-2↑, Bax↓, Caspase-3, cleaved-Caspase-3↓, PINK1↓, Parkin↓, p62↓, LC3-II↓, LC3-I↓, NADPH oxidase↓, iNOS↓, Drp1↓	Zhou et al. (2018)
Sodium tanshinone IIA sulfonate	*Salvia miltiorrhiza* Bunge (Danshen)	OGD/R cell (40 μmol/L)	Improve mitochondrial dysfunction, PP2A↑, Beclin 1↑, ATG5↑, p62↓, IL-10↑, TGF-β↑, BDNF↑, IL-1β↓, IL-2↓, TNF-α↓	Guo et al. (2023)
Trehalose	*saccharomyces*	SHRSP rats (2% aqueous solution, po, 3 months)	Mitophagy↑, Improve mitochondrial dysfunction, TNF-α↓, ROS↓, mtDNA↓, LC3↑, p62↑, TFEB↑	Wu et al. (2024)
Natural products target other mechanisms to treat stroke				
Baicalin	*Scutellaria baicalensis* Georgi	MCAO/R rats (100 mg/kg, once, at the onset of reperfusion) OGD/R PC12 cell (25 μM)	Mitochondrial division↓, Improve mitochondrial dysfunction, p-AMPK↑, p-Drp1↑, Drp-1↓, mmp↑, ROS↓, MFN2↑	Xie et al. (2023b)
Danhong injection	*Salvia miltiorrhiza* Bunge *Carthamus tinctorius* Linn.	MCAO/R rats (3 mL/kg, ip, twice/d, 14 d) OGD/R cell (0.1 μL/mL)	Improve mitochondrial dysfunction, parkin↑	Xue et al. (2023)
Ginsenoside Rb1	*Panax ginseng* C. A. Mey	MCAO/R mice (100 mg/kg, ip, once/d, 5 d) OGD/R cell (10 μM)	Mitochondrial transfer, Improve mitochondrial dysfunction, CD38↑, cADPR↑, ROS↓, Complex I↓, NADH↓, mmp↑, ATP↑	Li et al. (2022)
Ginkgolide K	*Ginkgo* L.	MCAO mice (8 mg/kg, ip, prior to the onset of reperfusion) OGD/R N2a cell (40 μM)	Mitochondrial division↓, mPTP↓, Improve mitochondrial dysfunction, Drp1↓, p-Drp1↑, GSK-3β↓, p-GSK-3β↑, ANT↑, CypD↓	He et al. (2023)

### Natural products targeting the mitochondrial apoptotic pathway for the treatment of stroke

**FIGURE 3:**
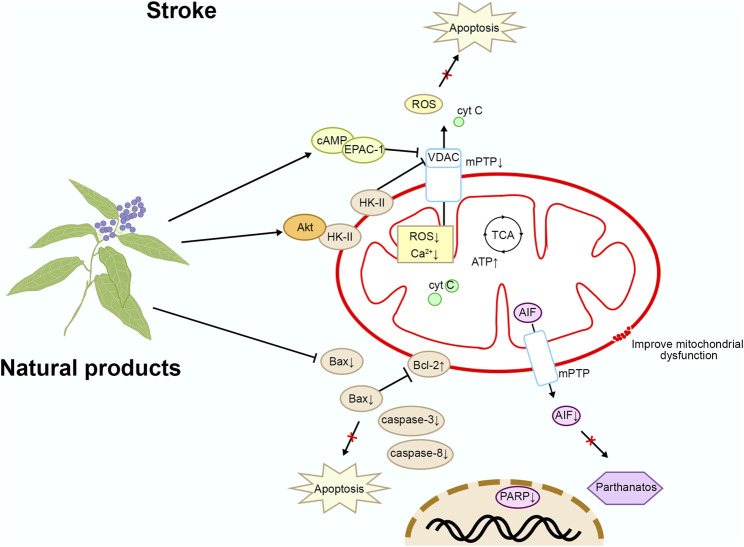
Mechanism of natural products targeting the mitochondrial apoptotic pathway for the treatment of stroke.

By intervening in the mitochondrial apoptosis pathway, neuronal apoptosis following stroke can be effectively reduced, protecting neurons from further damage and promoting the repair and regeneration of brain tissue (Xia et al., 2020). This therapeutic strategy not only alleviates neurological deficits in stroke patients but also improves their quality of life, potentially reducing disability and mortality rates after stroke. It holds significant importance for the rehabilitation and prognosis of stroke. Please refer to Figure 3 and Table 1 for details.

#### Astragaloside IV

Astragaloside IV (AIV) is a natural saponin extracted from *Astragalus membranaceus* Schischk., exhibiting broad biological activities. *In vitro* studies have found that glutamate stimulation induces the separation of HK-II from mitochondria, leading to mitochondrial dysfunction. Mitochondrial membrane damage leads to cell death through multiple mechanisms, including the activation of mPTP, increased ROS production, disruption of ATP synthesis, and induction of calcium overload, which collectively trigger apoptosis, necrosis, and inflammatory responses. After binding to the outer mitochondrial membrane, HK-II forms a complex with specific receptors on the membrane, thereby stabilizing the mitochondrial outer membrane structure and reducing cell death induced by membrane damage. AIV (purity ≥ 98%) at a concentration of 10 μM activates Akt by promoting its binding with HK-II, thereby protecting mitochondrial HK-II and preserving hexokinase activity by improving glycolysis. By retaining mitochondrial HK-II, AIV reduces the release of pro-apoptotic proteins and AIF, thus protecting neurons from apoptosis and Parthanatos. In addition, in middle cerebral artery occlusion/reperfusion (MCAO/R) mice, treatment with AIV at a dose of 30 mg/kg intraperitoneally for 7 days also activated Akt, promoted HK-II binding to mitochondria, and exerted a protective effect on neurons. In summary, AIV promotes the binding of HK-II with mitochondria via Akt, preserving the structural and functional integrity of mitochondria, thereby protecting neurons from apoptosis and DNA damage (Li et al., 2019). Furthermore, AIV inhibits the upregulation of Fas, FasL, Caspase-8, and Bax/Bcl-2 mRNA, as well as the protein levels of apoptosis-related factors Caspase-8, Bid, cleaved Caspase-3, and Cyto C after ischemia-reperfusion, suggesting that AIV may alleviate ischemia-reperfusion-induced apoptosis by inhibiting the activation of mitochondria-related apoptotic factors (Yin et al., 2020; Xue et al., 2019).

#### Celastrol

Celastrol is a natural product extracted from *Tripterygium wilfordii* Hook. f., known for its mitochondrial and neuronal protective effects. *In vivo* studies have shown that Celastrol at a dose of 2 mg/kg administered intraperitoneally once daily for 3 days improves neurological behavior and cognitive abilities in mice with intracerebral hemorrhage (ICH), reduces neuronal death, and promotes the recovery of neuronal mitochondrial function. *In vitro* research has revealed that Celastrol at a concentration of 10 μM binds with cyclic adenosine monophosphate (cAMP)/cAMP-activated exchange protein-1 (EPAC-1), inhibiting its interaction with voltage-dependent anion-selective channel protein 1 (VDAC1), thereby blocking the opening of the mPTP and reducing Primary Cortical Neuron death. Blocking the opening of mPTP effectively protects the integrity of the mitochondrial membrane, reduces ROS generation, and inhibits apoptosis and calcium overload, thereby preserving the healthy function of the mitochondria. In summary, Celastrol improves neuronal mitochondrial dysfunction induced by cerebral hemorrhage by targeting EPAC-1 (Li et al., 2024). In addition, Celastrol ameliorates cerebral ischaemia-reperfusion injury through antioxidant effects (Hong et al., 2023).

#### Crocetin

Crocetin is a natural compound derived from the herb *Crocus sativus* L., known for its antioxidant activity. *In vivo* studies on the permanent middle cerebral artery occlusion (MCAO) model in SD rats have shown that oral treatment with crocetin at a dose of 40 mg/kg once daily for 5 days inhibits the activation of NADPH oxidase 2 (NOX-2). This inhibition reduces the early production of ROS, PARP-1, poly (ADP-ribose) polymer (PAR), and apoptosis-inducing factor (AIF), thereby exerting neuroprotective effects in the brain. Additionally, PARylated hexokinase-I (HK-I) serves as a novel substrate of the E3 ubiquitin ligase RNF146. *In vitro* studies on SH-SY5Y cells induced by oxygen-glucose deprivation (OGD) have shown that Crocetin at a concentration of 25 μM inhibits RNF146-mediated HK-I degradation and ROS production, elevates mitochondrial membrane potential (mmp) and glutathione (GSH) levels, and reduces malondialdehyde (MDA) levels, thus preventing mitochondrial dysfunction and DNA damage. Moreover, Crocetin reduces the expression of p-p47, NOX-2, PARP-1, PAR, and AIF in cells, inhibiting neuronal parthanatos and protecting neuronal growth. NOX-2 is one of the key enzymes involved in oxidative stress, responsible for the generation of O₂⁻ and other ROS. During the occurrence of stroke, the excessive activation of NOX-2 leads to a substantial production of ROS, thereby exacerbating oxidative stress-induced mitochondrial damage, which ultimately results in brain tissue injury. These findings suggest that Crocetin exerts therapeutic effects against ischemic stroke by inhibiting NOX-2 and preventing HK-I degradation, thereby protecting mitochondrial function (Wu et al., 2023). In addition, Crocetin also prevents stroke by reducing ROS levels in the brain (Yoshino et al., 2011).

#### Oxyresveratrol

Oxyresveratrol is a natural compound extracted from various plants, such as *Veratrum album* L., known for its antioxidant and cardiovascular protective effects. *In vivo* studies have shown that Oxyresveratrol at a dose of 20 mg/kg injected intraperitoneally once a day for 2 days significantly reduces the infarct volume in MCAO rats and improves neurological deficits. Mechanistically, Oxyresveratrol reduces the release of cytochrome c and decreases caspase-3 activation in MCAO rats, thereby protecting mitochondrial function and reducing the number of apoptotic brain cells. Cytochrome c release and caspase-3 activation are central events in mitochondria-mediated apoptosis, and these processes result in rupture of the mitochondrial membrane, loss of function. Therefore, Oxyresveratrol exerts its anti-stroke effects by inhibiting the mitochondrial apoptotic pathway (Andrabi et al., 2004).

#### Picroside II

Picroside II is a natural compound extracted from *Neopicrorhiza scrophulariiflora* (Pennell), known for its anti-inflammatory and anti-apoptotic effects. *In vivo* studies have demonstrated that Picroside II (purity ≥ 98%) at a dose of 20 mg/kg administered by intraperitoneal injection 2 h after MCAO/R in rats reduces neuronal damage injury and improves the morphology of brain tissues. Mechanistically, Picroside II downregulates the expression of VDAC1, thereby reducing the permeability of the mitochondrial permeability transition pore (mPTP), decreasing ROS levels, and reducing the number of apoptotic cells. This inhibition suppresses the release of EndoG from mitochondria to the cytoplasm, thereby alleviating brain damage. Therefore, Picroside II mitigates ischemic brain injury by downregulating mitochondrial mPTP (Li et al., 2018; Zhang et al., 2017).

#### Theaflavic acid

Theaflavic acid (TFA) is a type of theaflavins found in black tea, exhibiting various biological activities. *In vitro* studies have revealed that 50 μM of TFA (purity ≥ 98%), targeting PC12 cells subjected to oxygen-glucose deprivation and reperfusion (OGD/R) injury, inhibits the excessive generation of intracellular ROS, reduces malondialdehyde levels, enhances superoxide dismutase activity, and improves cell viability while reducing lactate dehydrogenase (LDH) release to protect PC12 cells. Mechanistically, TFA inhibits intracellular calcium overload and mmp depolarization, decreases the expression of caspase-3 and Bax, and increases the protein expression of Bcl-2. Additionally, TFA promotes Nrf2 nuclear translocation, enhances ARE transcriptional activity, and upregulates HO-1 expression. These findings suggest that TFA mitigates OGD/R-induced neuronal cell damage by inhibiting the mitochondrial apoptotic pathway via the Nrf2/ARE signaling pathway (Li et al., 2020b; Mu et al., 2021).

#### TSG

2,3,5,4′-tetrahydroxystilbene-2-O-beta-D-glucoside (TSG) is a natural active component extracted from the root tuber of *Pleuropterus multiflorus* (Thunb.) Nakai, exhibiting antioxidant and anti-inflammatory effects. *In vivo* studies have shown that 40 mg/kg of TSG injected intraperitoneally prior to reperfusion significantly reduces cerebral infarction volume and neuronal apoptosis in rats subjected to MCAO. *In vitro* studies have demonstrated that 25 μM of TSG (purity ≥9 8%), targeting neuronal cells induced by OGD/R, reduces intracellular ROS and Ca2+ generation, decreases mmp, increases the ratio of Bcl-2/Bax, decreases the expression of p-JNK and iNOS, increases SIRT1 expression, and reduces neuronal damage. Therefore, TSG exerts neuroprotective effects by inhibiting mitochondria-related apoptosis (Wang et al., 2009).

### Natural products targeting mitochondrial oxidative stress for stroke treatment

**FIGURE 4:**
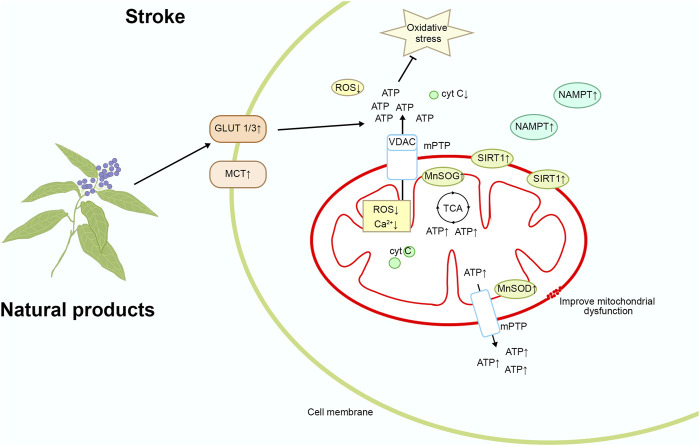
Mechanism of natural product targeting of mitochondrial oxidative stress for the treatment of stroke.

Stroke can lead to increased intracellular oxidative stress, resulting in mitochondrial dysfunction, mitochondrial DNA damage, and increased mitochondrial membrane permeability, thereby triggering apoptosis and inflammatory responses, exacerbating brain damage (Su et al., 2020). Targeting mitochondrial oxidative stress can alleviate oxidative stress damage to mitochondria, protect mitochondrial function, reduce apoptosis and inflammatory responses, help slow the progression of brain damage, and improve patient recovery and survival rates. Therefore, targeting mitochondrial oxidative stress is a promising therapeutic strategy that may bring new breakthroughs in the treatment of stroke. Please refer to Figure 4 and Table 1 for details.

#### Notoginsenoside R1

Notoginsenoside R1 is derived from the dried roots and rhizomes of *Panax notoginseng* (Burk.) F.H.Chen, exhibiting antioxidative, anti-inflammatory, anti-angiogenic, and anti-apoptotic activities. During ischemia, oxygen supply to the brain tissue is insufficient, and cells rely on anaerobic metabolism to produce lactate. Lactate is subsequently metabolized within the mitochondria and enters the TCA cycle, where it is further converted to ATP (Brooks, 2018). Simultaneously, brain cells depend on glucose as the primary substrate for ATP synthesis. *In vivo* studies have shown that treatment with 40 mg/kg of Notoginsenoside R1 injected intraperitoneally after MCAO reduces the infarct volume and neurological deficits in rats, increase ATP levels, and upregulate the expression of glucose transporter 1/3, monocarboxylate transporter 1, and citrate synthase in the peri-infarct tissue of the brain. *In vitro* studies have demonstrated that 20 μM of Notoginsenoside R1 (purity ≥ 98%) increases the number of mitochondria, mmp, and mitochondrial DNA copy number, thereby improving mitochondrial morphology and inhibiting Neuro-2a cell death induced by OGD. Additionally, the expression of mitochondrial energy metabolism-related mRNAs Atp12a and Atp6v1g3 is significantly upregulated. Therefore, Notoginsenoside R1 may improve neuronal mitochondrial function post-ischemic stroke and protect neurons from stroke-induced damage by enhancing brain glucose and lactate transport and ATP levels (Liu et al., 2022; Meng et al., 2014).

#### Notoginseng leaf triterpenes

Notoginseng leaf triterpenes (PNGL) are saponin compounds extracted from the roots and rhizomes of the plant *P. notoginseng* (Burk.) F. H. Chen, known for their therapeutic activity in cardiovascular diseases. *In vivo* studies have shown that treatment with PNGL at a dose of 292 mg/kg by gavage once daily for 2 weeks significantly alleviates oxidative stress, inhibits mitochondrial damage, mitigates energy metabolism dysfunction, reduces neuronal loss and apoptosis, thereby markedly improving the survival rate of neurons under ischemic and hypoxic conditions in the MCAO/R model. Additionally, PNGL significantly increases the expression of nicotinamide phosphoribosyltransferase (NAMPT) in the ischemic area, thereby regulating its downstream pathways involving SIRT1/2/3-MnSOD/PGC-1α (Xie W. et al., 2023). *In vitro* studies have revealed that PNGL at a concentration of 6.25 μg/mL markedly alleviates ischemic damage, maintains redox balance and alleviates oxidative stress, inhibits mitochondrial damage, mitigates energy metabolism dysfunction, improves neuronal mitochondrial function, and significantly reduces neuronal loss and apoptosis in SH-SY5Y cells subjected to OGD/R. Moreover, PNGL significantly increases NAMPT expression in OGD/R cells and activates downstream pathways involving SIRT1/2-Foxo3a and SIRT1/3-MnSOD/PGC-1α. Ischemic events are typically accompanied by oxidative stress and dysregulated cellular energy metabolism. NAMPT promotes an increase in intracellular NAD⁺ levels, which activates SIRT1. SIRT1 primarily exerts its effects through deacetylation, regulating various transcription factors such as NF-κB and p53, thereby inhibiting the expression of oxidative stress-related genes (Yan et al., 2021). Additionally, SIRT1 enhances the activity of antioxidant enzymes, such as MnSOD, CAT, and GPX, reducing the generation of free radicals and ROS, ultimately alleviating oxidative damage (Bai et al., 2016). In summary, PNGL exerts mitochondrial protective effects and holds promise as a treatment for stroke by acting through NAMPT (Xie et al., 2020).

#### Panax notoginseng saponins

Panax notoginseng saponins (PNS) are natural compounds extracted from the roots and rhizomes of the plant *P. notoginseng* (Burk.) F. H. Chen, known for their therapeutic activity in cardiovascular diseases. *In vivo* studies have shown that oral treatment with PNS (purity > 95%) at a dose of 60 mg/kg once daily for 6 months significantly improves the morphological changes in the myocardium of aging rats, increases the ratio of Bcl-2/Bax and decreases the protein expression of caspase-3, thereby preventing an increase in myocardial cell apoptosis, reducing fractured mitochondrial cristae, and ameliorating age-related mitochondrial dysfunction. PNS also significantly reverses the downregulation of FoxO3a and Mn-SOD and the upregulation of PGC-1α, LC3β, and Beclin-1 levels. FoxO3a is involved in cellular antioxidant responses and stress reactions. Mn-SOD, a key antioxidant enzyme in mitochondria, functions to eliminate O₂⁻, thereby reducing the accumulation of ROS. LC3β and Beclin-1 are critical marker proteins in the autophagy process, playing essential roles in the formation of autophagosomes. During the aging process, mitochondrial dysfunction leads to increased oxidative damage, which plays a crucial role in myocardial cell apoptosis. In summary, PNS alleviates oxidative damage through oxidative stress and mitochondria-related signaling pathways, exerting an anti-myocardial cell apoptosis effect and potentially reducing the occurrence of stroke (Zhou et al., 2018).

### Natural product targeting of mitochondrial iron death for the treatment of stroke

**FIGURE 5:**
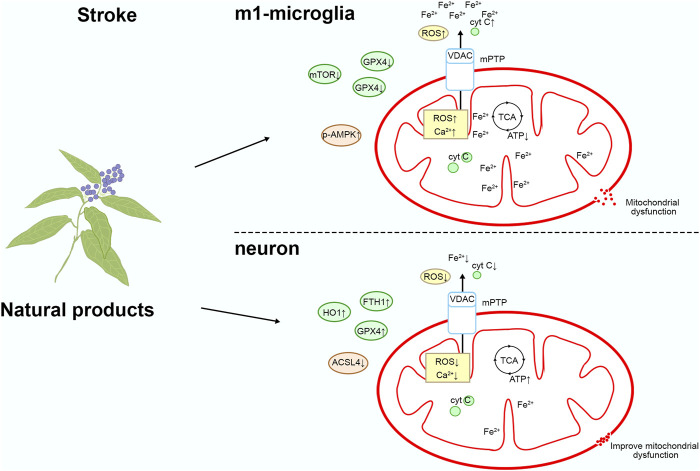
Mechanism of natural product targeting of mitochondrial iron death for the treatment of stroke.

During the process of stroke, impaired blood perfusion leads to cerebral hypoxia-ischemia, triggering the release of iron ions and intracellular iron overload, consequently activating oxidative stress responses and the iron death pathway. Iron death accelerates apoptosis of brain cells and inflammatory reactions, exacerbating the extent of brain damage (Guo et al., 2023). Therefore, intervention in the iron death pathway may help alleviate the damage caused by stroke and play a crucial role in the treatment and prevention of strokes. Please refer to Figure 5 and Table 1 for details.

#### Artesunate

Artesunate is a water-soluble semi-synthetic derivative of artemisinin, derived from *Artemisia caruifolia* Buch.-Ham., exhibiting significant anti-neuroinflammatory pharmacological effects. In some cases, iron death may help remove damaged or abnormal cells, preventing them from further damaging surrounding healthy neurons (Wu et al., 2024). In neuroinflammatory responses, removal of damaged cells may reduce the release of inflammatory factors, thereby mitigating further nerve damage. *In vivo* studies have revealed that in rats with intracerebral hemorrhage (ICH) injury, intraperitoneal injection of 70 mg/kg of artesunate once daily for 3 days improves neurological deficits, reduces hematoma volume, and alleviates brain edema. Additionally, artesunate inhibits pro-inflammatory factors associated with M1-type microglia and upregulates iron death. *In vitro*, treatment with 10 μM of artesunate upregulates ROS and lipid peroxidation levels, activates p-AMPK, inhibits the expression of mTORC1, p-Akt, and GPX4, and reduces the viability of BV2 cells stimulated by LPS. Therefore, artesunate induces iron death in M1-polarized microglia, inhibits inflammation, and alleviates secondary damage caused by cerebral hemorrhage (Xie G. et al., 2023).

#### Baicalein

Baicalein is the primary bioactive component isolated from the roots of *Scutellaria baicalensis* Georgi. *In vivo* studies have shown that intraperitoneal administration of 80 mg/kg baicalein (purity > 98%) once daily for 3 days to MCAO mice ameliorated cerebral I/R injury by decreasing brain tissue Fe2+ levels, lipid peroxidation, and the characteristic morphological features of iron death in mitochondria. *In vitro* studies on HT22 cells subjected to OGD/R reveal that 4 μM of baicalein inhibits cellular iron death by promoting the levels of GPX4 and ACSL3 while suppressing the expression of ACSL4. GPX4 converts lipid peroxides into their corresponding alcohols, preventing the further accumulation of peroxides, thereby reducing oxidative stress and protecting cells from ferroptosis (Xue et al., 2023). ACSL4 is closely involved in the regulation of lipid metabolism, and increased expression of ACSL4 promotes the synthesis of polyunsaturated fatty acids, making cell membrane lipids more prone to oxidation, thereby enhancing sensitivity to ferroptosis (Xue et al., 2023). Consequently, baicalein inhibits iron death and alleviates damage caused by stroke (Li et al., 2022).

#### Cottonseed oil

Cottonseed oil (CSO) is a vegetable oil extracted from *Gossypium hirsutum* Linn., containing a high concentration of essential fatty acids for human consumption. In MCAO-R-induced rats, subcutaneous injections using 1.3 mL/kg CSO (purity > 99%) every 2 days for 3 weeks resulted in reductions in infarct area and neuronal damage, significantly improving rat neurological dysfunction. Mechanistically, CSO upregulates the expression of anti-iron death proteins (GPX4, xCT, HO1, FTH1) while downregulating the expression of iron death-related protein ACSL4. This leads to increased activity of GSH and SOD, and decreased levels of MDA and LPO, thereby alleviating mitochondrial damage caused by ischemic stroke. xCT, HO1, and FTH1 regulate ferroptosis through distinct mechanisms. Upregulation of xCT increases GSH levels, alleviating oxidative stress and thereby delaying the onset of ferroptosis (He et al., 2023). The product of HO1, bilirubin, possesses potent antioxidant properties, scavenging free radicals and mitigating oxidative damage, thus inhibiting iron overload-induced cellular damage and ferroptosis (Tang et al., 2021). FTH1, on the other hand, functions by binding and storing free iron, preventing oxidative damage caused by free iron (Kong et al., 2021). Consequently, CSO treatment can mitigate ischemic stroke injury by inhibiting iron death (Sun et al., 2023).

### Natural product-targeted mitochondrial autophagy for stroke treatment

**FIGURE 6:**
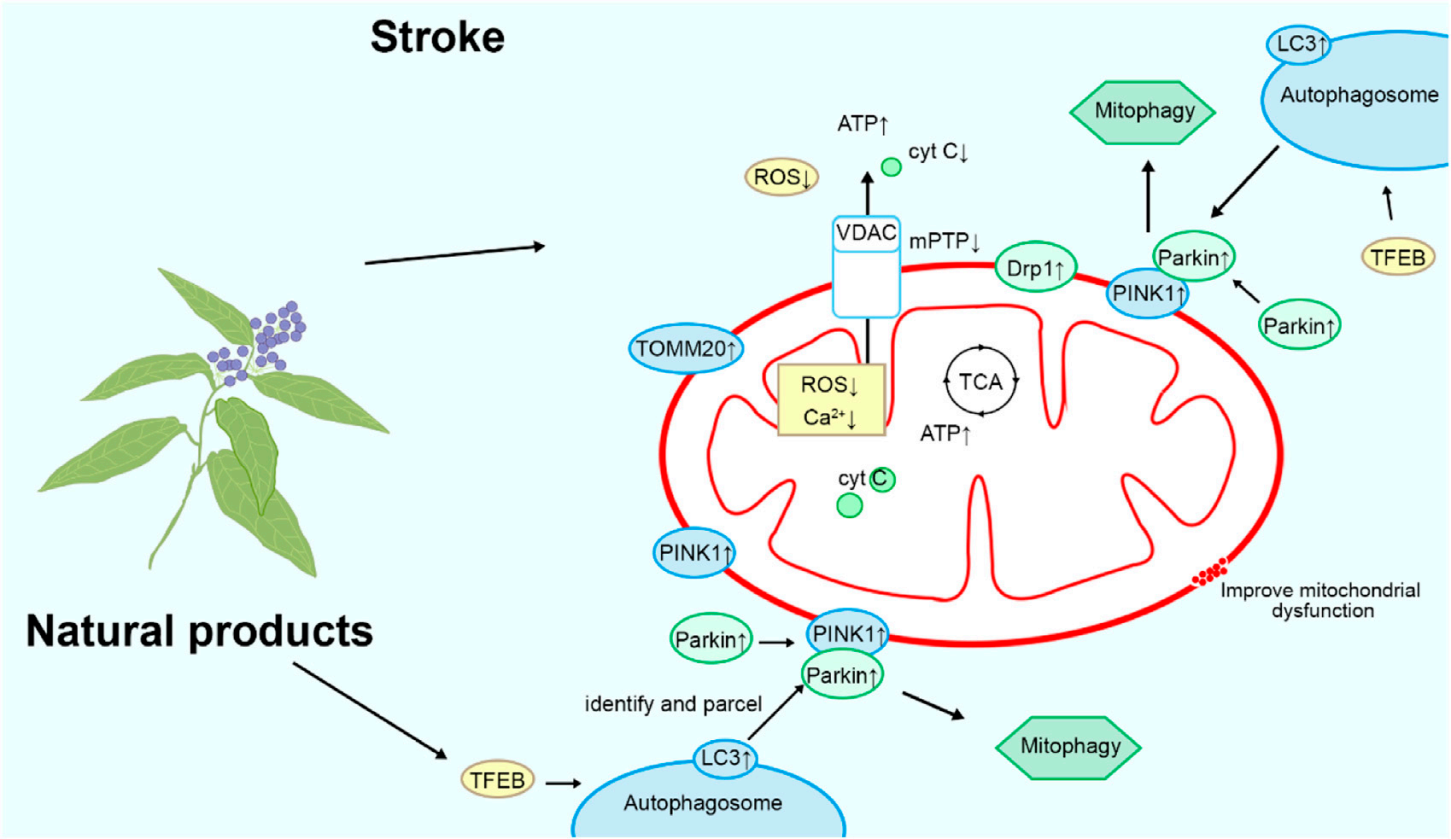
Mechanism of natural products targeting mitochondrial autophagy for the treatment of stroke.

Mitochondrial autophagy contributes to the clearance of dysfunctional mitochondria and damaged organelles, alleviating cell damage and inflammatory responses induced by oxidative stress, thereby protecting neurons from further harm (Li J. et al., 2023). Additionally, mitochondrial autophagy helps maintain cellular energy metabolism balance, promoting cell survival and repair, thus offering potentially robust support for stroke recovery. Please refer to Figure 6 and Table 1 for details.

#### Kaempferol

Kaempferol is a natural flavonoid compound found in most plants, such as *Brassica capitata* var. italica. It exhibits various biological activities, including anticancer and anti-inflammatory effects. *In vitro* studies have shown that oral administration of 10 μM of kaempferol (purity ≥ 98%) once daily for 7 days targets OGD neuron cells, activating Akt in OGD-treated neuronal cells, maintaining mitochondrial HK-II levels and activating dynamin-related protein 1 (Drp1), which regulates mitochondrial dynamics. This process inhibits excessive mitochondrial fission, reducing mitochondrial damage and thus protecting neurons (Schmitt et al., 2018). Kaempferol also activates LC3, promoting mitophagy. Mitophagy helps clear damaged and dysfunctional mitochondria, preventing them from inducing oxidative damage and cell death (Towers et al., 2021). *In vivo* studies targeting MCAO rats have demonstrated that 200 mg/kg of kaempferol can reduce the infarct volume in rats and activate HK-II and Drp1 in the infarct area, reproducing similar mitochondrial protective effects. Therefore, kaempferol exerts its therapeutic effects on stroke by promoting mitochondrial autophagy (Wu et al., 2017).

#### Ligustilide

Ligustilide is a natural compound extracted from *Angelica sinensis* (Oliv.) Diels, known for its neuroprotective properties. *In vivo* studies on MCAO/R rats showed disordered arrangement of hippocampal neurons, disappearance of nucleoli, and shrinkage of nuclear plasm. Intraperitoneal injection of 20 mg/kg of ligustilide (purity: 99.17%) once a day for 3 days increased the expression of PINK1, Parkin, TOMM20, and LC3, enhancing mitochondrial autophagy, improving mitochondrial function, and alleviating ischemia-reperfusion injury in rat brains. *In vitro* studies targeting OGD/R-induced HT-22 cells demonstrated that 20 μM of ligustilide promoted mitochondrial autophagy by upregulating PINK1/Parkin, reducing ROS levels, protecting mitochondrial function, and ameliorating neuronal damage caused by ischemic stroke. Overall, ligustilide exerts its protective effects against stroke by promoting mitochondrial autophagy (Wu et al., 2022; Mao et al., 2022).

#### Quercetin

Quercetin is a natural flavonoid compound widely distributed in various plants, such as *Glycyrrhiza uralensis* Fisch., known for its antioxidant, anticancer, hypoglycemic, and hypolipidemic properties. *In vitro* studies targeting primary rat brain microvascular endothelial cells (BMECs) showed that the combination of 200 μM quercetin and hyaluronic acid activated LC3, PINK1, and TFEB, promoting mitochondrial autophagy, reducing ROS levels, and decreasing endothelial cell apoptosis. *In vivo* experiments on MCAO rats demonstrated that tail vein injection of 5 mg/kg quercetin and hyaluronic acid 2 h after surgery inhibited the protein expression of p-Akt, p-mTOR, HIF-1α, and LC3, increased the protein levels of PINK1 and Parkin, activated mitochondrial autophagy, exerted neuroprotective effects, and reduced the infarct area. In summary, quercetin treats ischemic brain injury by activating mitochondrial autophagy (Cen et al., 2022).

#### Rehmapicroside

Rehmapicroside is a rosmarinic acid glycoside isolated from the rhizomes of *Rehmannia glutinosa* (Gaert.) Libosch, known for its cardiocerebrovascular protective effects. ONOO⁻ is a highly oxidative molecule typically generated during ischemia and reperfusion through the reaction between NO and O₂⁻ (Zhang et al., 2023). During ischemia-reperfusion injury, the oxygen supply is insufficient due to ischemia, leading to the generation of a large amount of ROS inside the cells. These ROS react with NO, resulting in the production of abundant ONOO⁻. *In vitro* studies have shown that during ischemia-reperfusion, ONOO⁻ exerts its effects by recruiting Drp1 to the mitochondria, causing excessive mitochondrial fission and generating more damaged mitochondria. In PC12 cells induced by OGD/R, 50 μM rehmapicroside (purity ≥ 98%) reduced O_2_^−^ and ONOO^−^, upregulated Bcl-2, downregulated Bax, Caspase-3, and cleaved Caspase-3 expression, and decreased the ratio of LC3-II to LC3-I along with downregulation of PINK1, Parkin, p62, and LC3-II levels, thereby inhibiting mitochondrial-related apoptotic pathways and autophagy, ultimately protecting neuronal viability. *In vivo* studies on MCAO/R rats have shown that intraperitoneal injection of 10 mg/kg rehmapicroside at the onset of reperfusion improved infarct area and neurofunctional deficits. Mechanistically, rehmapicroside inhibited 3-nitrotyrosine formation, NADPH oxidase, and iNOS expression, while preventing PINK1, Parkin, and Drp1 translocation into mitochondria to activate mitochondrial autophagy. Therefore, rehmapicroside treats cerebral ischemia by inhibiting excessive mitochondrial autophagy (Zhang et al., 2020).

#### Sodium tanshinone IIA sulfonate

Sodium tanshinone IIA sulfonate (STS) is a water-soluble derivative of tanshinone IIA, the main bioactive component extracted from the root of *Salvia miltiorrhiza* Bunge (Danshen), known for its anti-inflammatory and cardiocerebrovascular protective effects. *In vitro* studies have shown that in small glial cells and neurons induced by OGD/R, 40 μmol/L of STS significantly upregulates the expression of PP2A in small glial cells, leading to increased levels of Beclin 1 and ATG5 and decreased levels of the p62 protein, inducing cell autophagy. STS-induced autophagy in small glial cells reduces the production of anti-inflammatory factors (IL-10, TGF-β, and BDNF) and induces the release of pro-inflammatory factors (IL-1β, IL-2, and TNF-α), thereby inhibiting neuronal mitochondrial dysfunction and apoptosis and exerting a protective effect on neurons. In summary, STS regulates autophagy and inflammation in small glial cells through the PP2A gene, improving mitochondrial function, and inhibiting neuronal apoptosis (Ma et al., 2023).

#### Trehalose

Trehalose is a naturally occurring non-reducing disaccharide synthesized by lower organisms such as yeast and slow-moving animals. It exhibits antioxidant properties, reduces protein aggregation, and enhances autophagy. *In vivo* studies have found that feeding spontaneously hypertensive stroke-prone (SHRSP) rats with a high-salt stroke-prone diet (JD) supplemented with a 2% concentration of trehalose orally for 3 months reduces the incidence of stroke. At the molecular level, trehalose upregulates the expression of LC3 and p62, inhibits TNF-α activation and ROS production, reduces mtDNA content, upregulates TFEB expression, promotes its nuclear translocation, and enhances mitochondrial autophagy, improving mitochondrial function. These results indicate that trehalose exerts its anti-stroke effects by promoting mitochondrial autophagy (Forte et al., 2021).

### Other mechanisms of natural product targeting of mitochondria for the treatment of stroke

#### Baicalin

Baicalin is a flavonoid natural compound extracted from *S. baicalensis* Georgi, known for its neuroprotective activity. High blood sugar is a risk factor for exacerbating brain defects. *In vivo* studies have found that 100 mg/kg of baicalin in MCAO rats can reduce blood sugar, alleviate neurological deficits, and decrease infarct volume. *In vitro* studies have shown that 10 μM of baicalin (purity ≥ 98%) in PC12 cells induced by OGD/R inhibits Drp-1 expression, reduces mitochondrial fission, promotes the generation of mitofusin-2 (MFN2), increases Drp-1 Ser637 phosphorylation, and enhances mmp by inhibiting ROS production. Knockdown of AMPKα1 abolishes the protective effect of baicalin. Baicalin also inhibits cell apoptosis and enhances autophagy. These results suggest that baicalin protects against exacerbated brain ischemic damage induced by high blood sugar by regulating mitochondrial function via AMPK (Li et al., 2017).

#### Danhong injection

Danhong Injection (DHI) is a natural medicine extracted from *S. miltiorrhiza* Bunge and Carthamus tinctorius Linn., known for its blood-activating and stasis-removing effects. *In vitro* studies have shown that 0.1 μL/mL of DHI on OGD/R neurons upregulates the expression of the Parkin protein, regulates mitochondrial dynamics, suppresses mitochondrial damage, and enhances neuronal activity. *In vivo* studies on MCAO/R rat models have demonstrated that intraperitoneal injection of 3 mL/kg of DHI twice a day for 14 days increases Parkin protein levels, repairs nerve defects caused by cerebral ischemia and sensory-motor impairment, and improves rat survival rates. In summary, DHI exerts its anti-stroke effects by protecting mitochondrial function through Parkin (Orgah et al., 2019).

#### Ginsenoside Rb1

Ginsenoside Rb1, extracted from *Panax ginseng* C. A. Mey, is a natural saponin compound with neuroprotective potential. In murine MCAO/R models, intraperitoneal injection of rb1 at a dosage of 100 mg/kg once daily for 5 days inhibits ROS production in a CD38-dependent manner. It catalyzes the synthesis of cyclic ADP-ribose (cADPR), a calcium messenger, on the mitochondrial membrane to promote the transfer of astrocytic mitochondria to neurons, thereby protecting neuronal survival and alleviating ischemic brain injury. In OGD/R models, 10 μM of Rb1 inhibits the activation of NADH dehydrogenase in mitochondrial complex I, blocking the generation of ROS from reverse electron transport in complex I. This action deactivates astrocytes to protect mitochondria. When neurons are damaged by OGD/R, Rb1 protects astrocytic mitochondria and promotes their transfer, thereby increasing mmp and ATP production to protect neurons. Thus, the transfer of astrocytic mitochondria appears to be a mechanism by which Rb1 promotes neuronal survival and function (Ni et al., 2022).

#### Ginkgolide K

Ginkgolide K is a natural compound extracted from the leaves of *Ginkgo* L., possessing multiple pharmacological activities. *In vivo* studies have found that in mice induced with MCAO, intraperitoneal injection of ginkgolide K at a dose of 8 mg/kg prior to the onset of reperfusion inhibits the translocation of GSK-3β and Drp1 to mitochondria, alleviating mitochondrial dysfunction. *In vitro* studies targeting N2a cells induced with OGD/R revealed that 40 μM of ginkgolide K increases phosphorylation at the Ser637 site of Drp1, suppressing the recruitment of Drp1 to mitochondria and thereby reducing mitochondrial fission. Moreover, ginkgolide K induces phosphorylation at the Ser9 site of GSK-3β, enhancing the interaction between adenosine nucleotide transporter (ANT) and p-GSK-3β. This interaction inhibits the binding of ANT with cyclophilin D (CypD), thereby suppressing the opening of mitochondrial permeability transition pores (mPTP). Thus, ginkgolide K protects mitochondrial function and exerts anti-stroke effects by inhibiting mitochondrial fission and mPTP (Zhou et al., 2017).

In summary, current research indicates that natural products demonstrate tremendous potential in targeting mitochondrial protection against neuronal damage, further advancing the possibilities for stroke treatment. These studies have revealed that most natural products play a crucial role in protecting neuronal mitochondria by modulating the expression of various mitochondrial-related proteins, such as Bax, Bcl-2, caspases-3/-8, Drp-1, PINK1, and Parkin. Additionally, natural products can directly enhance mmp, inhibit the opening of mPTP, reduce ROS generation, promote mitochondrial autophagy and transfer, and suppress mitochondrial fission, thereby safeguarding neurons from injury. These research findings provide important theoretical and experimental foundations for developing novel stroke treatment strategies, paving the way for further exploration of the potential of natural products in stroke therapy.

In addition, compared to synthetic drugs, natural products offer significant advantages, including higher safety, favorable bioavailability, and multi-target effects (Katz and Baltz, 2016). Natural products, which are typically derived from plants, animals, or microorganisms, have been proven through long-term traditional use to possess lower toxicity and fewer side effects, making them safer for clinical application. They generally exhibit better absorption and metabolic characteristics in the body, leading to higher bioavailability. Furthermore, because natural compounds contain multiple bioactive components, they can exert effects on various biological targets through different mechanisms, thereby enhancing therapeutic efficacy through synergistic actions (Kalkreuter et al., 2020). These properties make natural products particularly promising for the treatment of complex diseases, especially in the management of neurodegenerative conditions such as stroke.

Although these natural compounds have shown potential efficacy in the treatment of stroke, particularly in alleviating oxidative stress, inhibiting inflammation, protecting neurons, and promoting mitochondrial function recovery, their clinical application still faces several challenges and limitations. First, most existing studies are focused on *in vitro* and animal models, and there is a lack of sufficient large-scale, randomized controlled clinical trials to verify their efficacy and safety. Second, the mechanisms of action and optimal administration routes for different natural compounds remain unclear, and their pharmacokinetic properties and bioavailability need further investigation. Additionally, the long-term efficacy, safety, potential drug interactions, and side effects of these natural compounds have not been thoroughly evaluated. Therefore, while natural compounds show promise in stroke treatment, future research should focus on clinical validation, dosage optimization, personalized treatment strategies, and the safety of long-term use to ensure their effectiveness and feasibility in stroke therapy.

## Clinical application and research progress of natural products in stroke treatment

The clinical application and research progress of natural products in stroke have garnered significant attention (Tao et al., 2020). Currently, numerous natural products are being used in clinical therapy for stroke. For instance, Neuroaid has been shown to significantly reduce vascular risks and early vascular events in stroke patients, with a longer-lasting therapeutic effect (Chen et al., 2013; Suwanwela et al., 2018). Tongxinluo, a traditional Chinese medicine compound, can decrease adverse cardiovascular and cerebrovascular events within 30 days in patients (Yang et al., 2023). The omega-3 fatty acids eicosapentaenoic acid (EPA) and docosahexaenoic acid (DHA) can notably reduce the risk of stroke in patients (Nicholls et al., 2020). Saffron significantly reduces the severity of ischemic stroke, while elevating levels of glutathione (GSH) and total antioxidant capacity (TAC), and decreasing levels of malondialdehyde (MDA) (Gudarzi et al., 2022).

Some monomeric compounds found in natural products have garnered significant attention in the field of stroke treatment and are widely researched. Apart from their demonstrated efficacy in clinical trials, these compounds also exhibit remarkable properties at the molecular biology level. For instance, resveratrol, curcumin, ginsenosides, among others, play crucial roles by modulating inflammatory signaling pathways, alleviating oxidative stress, and inhibiting cell apoptosis (Hou et al., 2018; Wu et al., 2021; Chu et al., 2019). Molecular-level studies have indicated that these compounds can intervene in multiple cellular signaling pathways, including the JAK2/STAT3 and Nrf2/ARE signaling pathways, to repair the blood-brain barrier, promote neuronal survival and repair, thereby offering new directions and strategies for stroke treatment. Thus, combining clinical trials with molecular biology research, the monomeric compounds in these natural products hold potential therapeutic effects, bringing new hope for stroke patients.

Compared to synthetic drugs, natural products have a broader range of sources and a more diverse chemical structure, thus possessing greater potential pharmacological activity and biological effects (Fan et al., 2022; Karageorgis et al., 2021; Chen et al., 2020). These natural products include, but are not limited to, plant extracts, marine biota components, and microbial metabolites, which have gradually developed unique biological activities through long-term evolution in natural environments (Sflakidou et al., 2022; Xu et al., 2020). These preclinical studies not only confirm the neuroprotective effects of natural products in stroke treatment but also provide a more solid scientific foundation for their clinical application. Therefore, natural products, due to their natural, safe, and effective characteristics, have become an important resource attracting considerable attention in the field of stroke treatment.

## Conclusions and future research directions and perspectives

Based on recent research findings, this paper focuses on summarizing a series of naturally occurring compounds with anti-stroke activity, along with their sources, structural classifications, and mechanisms of action. These natural compounds are derived primarily from plants and microbial metabolites, with terpenoids, alkaloids, glycosides, and flavonoids being the main classes. Through comparative analysis of the mechanisms of action of these compounds, it is found that they exhibit various mechanisms, including regulating the expression of mitochondrial-related apoptotic proteins (such as Bcl-2, Bax, caspase-3, etc.), inhibiting ROS generation, regulating ferroptosis, promoting mitochondrial autophagy and transfer, among others.

The comprehensive review suggests that these natural products can exert neuroprotective effects by modulating mitochondrial function, although the conclusions are still preliminary. Nano-delivery systems and synthetic biology can also provide effective solutions for the clinical application of natural products. The former solves the problem of delivery efficiency by improving bioavailability, stability and targeting, while the latter improves the efficacy and safety of natural products by optimising production processes and structural modifications (Parodi et al., 2017). Therefore, it is recommended to select natural products that have beneficial effects on mitochondria and their metabolism, further conduct structural modification and derivative development based on their natural structures, and determine the optimal effective dose for stroke through *in vitro* and *in vivo* experiments. In addition, further optimization of formulations and administration methods is needed to minimize potential toxic side effects on healthy cells, develop safer formulations. In the future, this natural product treatment model may become a promising and safer approach for stroke therapy.
